# CTPA with a conventional CT at 100 kVp vs. a spectral-detector CT at 120 kVp: Comparison of radiation exposure, diagnostic performance and image quality

**DOI:** 10.1016/j.ejro.2020.100234

**Published:** 2020-05-07

**Authors:** Andreas P. Sauter, Nadav Shapira, Felix K. Kopp, Juliane Aichele, Jannis Bodden, Andreas Knipfer, Ernst J. Rummeny, Peter B. Noël

**Affiliations:** a Technical University of Munich, School of Medicine, Klinikum rechts der Isar, Department of Diagnostic and Interventional Radiology, Munich, Germany; b Department of Radiology, Perelman School of Medicine, University of Pennsylvania, Philadelphia, USA; c Philips Healthcare, Haifa, Israel

**Keywords:** BMI, body mass index, CNR, contrast-to-noise ratio, CT, computed tomography, C-CT, conventional spiral CT, CTDI_VOL_, volume-weighted CT dose index, CTPA, CT pulmonary angiography, DE-CT, dual-Energy CT, DS-CT, dual-Source CT, DLP, dose length product, ED, effective dose, IQ, image quality, keV, kilo-electronvolt, kVp, peak kilovoltage, HU, Hounsfield Units, PE, pulmonary embolism, ROI, region of interest, SD-CT, spectral-detector CT, VMI, virtual monochromatic images, Pulmonary embolism, Computed tomography angiography, Radiation exposure, Technology, Radiologic, Patient safety

## Abstract

**Purpose:**

To compare CT pulmonary angiographies (CTPAs) as well as phantom scans obtained at 100 kVp with a conventional CT (C-CT) to virtual monochromatic images (VMI) obtained with a spectral detector CT (SD-CT) at equivalent dose levels as well as to compare the radiation exposure of both systems.

**Material and Methods:**

In total, 2110 patients with suspected pulmonary embolism (PE) were examined with both systems. For each system (C-CT and SD-CT), imaging data of 30 patients with the same mean CT dose index (4.85 mGy) was used for the reader study. C-CT was performed with 100 kVp and SD-CT was performed with 120 kVp; for SD-CT, virtual monochromatic images (VMI) with 40, 60 and 70 keV were calculated. All datasets were evaluated by three blinded radiologists regarding image quality, diagnostic confidence and diagnostic performance (sensitivity, specificity). Contrast-to-noise ratio (CNR) for different iodine concentrations was evaluated in a phantom study.

**Results:**

CNR was significantly higher with VMI at 40 keV compared to all other datasets. Subjective image quality as well as sensitivity and specificity showed the highest values with VMI at 60 keV and 70 keV. Hereby, a significant difference to 100 kVp (C-CT) was found for image quality. The highest sensitivity was found using VMI at 60 keV with a sensitivity of more than 97 % for all localizations of PE. For diagnostic confidence and subjective contrast, highest values were found with VMI at 40 keV.

**Conclusion:**

Higher levels of diagnostic performance and image quality were achieved for CPTAs with SD-CT compared to C-CT given similar dose levels. In the clinical setting SD-CT may be the modality of choice as additional spectral information can be obtained.

## Highlights

• With SD-CT, increased radiation exposure is not present.
• In the current study, CTDI_vol_ was lower with SD-CT than with C-CT, even when 100 kVp was used for the latter.
• With SD-CT, higher levels of diagnostic performance and image quality can be achieved.
• SD-CT may be the system of choice due to the availability of spectral data and thus additional image information.

## 1 Introduction

Computed tomography (CT) is a standard imaging modality with a high importance in various clinical fields. Most CT examinations are performed with intravenously applied contrast agent to increase the contrast of vessels and organs and thus the detectability of pathologies [1]. All routinely used contrast agents for CT diagnostics contain iodine as the attenuating material [1]. With conventional CT systems, iodine can only be quantified indirectly by comparing unenhanced and enhanced scans or by measuring the absorption of x-rays via Hounsfield Units (HU). However, attenuation can be influenced by the density and the composition of the examined material as well as by the iodine uptake after application of contrast medium. Thus, iodine cannot be directly differentiated or quantified with conventional CT systems [2]. This drawback has been overcome by dual-energy CT (DE-CT) [3]. With these systems, two different energy spectra are used for the differentiation and quantification of two materials [4]. In clinical routine, iodine is distinguished from other materials and is then quantified [5] or subtracted [6]. There are different approaches to obtain the needed two energy spectra, such as dual-source CT (DS-CT), rapid-kVp switching CT and split-beam CT [[7], [8], [9]]. Another approach is the usage of a consistent tube voltage (120 kVp) in a spectral detector CT (SD-CT). This system uses a detector which consist of two layers. The upper layer absorbs low-energy photons and is transparent for high-energy photons which are absorbed by the lower layer [10]. Low- and high-energy images are generated and full spectrum images with spectral information are calculated by weighted summation. With other DE-CT systems, examinations must be planned to obtain spectral data before beginning of the CT scan. In contrast, SD-CT systems automatically acquire spectral data in each scan without modification of scanning parameters. Thus, a full retrospective spectral evaluation is possible whereas with other DE-CT systems, a prospective selection of patients for DE-CT is required. This is helpful when a non-enhanced contrast phase is needed for diagnostics but was not obtained initially, e.g. in case of incidental findings. Another advantage of SD-CT is the full temporal and spatial alignment [11]. Furthermore, all techniques for reduction of radiation dose (e.g. limiting the field-of-view or reducing the gantry rotation time) can be applied for SD-CT [11]. Radiation exposure of CT examinations is a concern due to the increased lifetime risk of malignancies [12]. As a sufficient radiation dose is needed for the accurate quantification of materials like iodine [13,14], concerns about radiation exposure of DE-CT were raised. For DS-CT and rapid kVp-switching systems, studies showed different results regarding radiation dose depending on the clinical application, system preferences and patient type [[15], [16], [17], [18], [19]]. For SD-CT, studies showed identical or lower radiation exposure compared to conventional CT systems; however, only one patient study exists for this purpose [20,21].

For all clinically available DE-CT systems, multiple usages for spectral data exist, e.g. the detection and differentiation of tumors such as head and neck tumors, renal lesion, pulmonary lesions or pancreatic adenomas [[22], [23], [24], [25], [26], [27]]. One important application is the calculation of virtual monochromatic images (VMI). In VMI, spectral data is used to calculate images as if a single tube voltage (e.g. 40 kVp) was used. With VMI at low keV-levels, the iodine signal is boosted due to the high photoelectric component (Z_Iodine_ = 53) as well as due to the proximity to the k-edge of iodine at 33 kVp [28,29]. This is especially useful in high contrast examinations like CT pulmonary angiography (CTPA) or imaging of the aorta [30,31]. With conventional CT systems, a similar effect can be achieved by choosing a lower tube voltage (increasing the k-edge effect and the contribution of the photoelectric effect), e.g. 70–100 kVp instead of 120 kVp [[32], [33], [34], [35]]. Due to an increased attenuation of iodine, a dose reduction can be possible for high-contrast examinations in normal-weighted patients [36]. However, examinations with such tube voltages can only be performed in patients with a normal bodyweight or in brain imaging as in obese patients a reduction of the tube voltage is not possible due to photon starvation. These limitations are overcome with SD-CT as VMI can be generated from the spectral data and the conventional CT images with a usual image appearance are always available for comparison. Thus, one has not to choose between an increased attenuation of iodine and conventional imaging of the other structures. To the best of our knowledge, to date there are no studies comparing examinations at 100 kVp to VMI of SD-CT. In the current study, CTPAs of patients with suspected pulmonary embolism were compared as this examination can be performed with a tube voltage of 100 kVp. Thus, a comparison of 100 kVp images and VMI images of SD-CT at 120 kVp was possible. As additional applications such as iodine quantification and iodine enhancement as well as material decomposition are available with SD-CT, this system could be superior to conventional CT if similar dose levels and image quality would be maintained.

The aim of the current study was to compare CTPAs and phantom scans obtained with 100 kVp to VMI (40, 60, 70 keV) obtained with a SD-CT using 120 kVp at equivalent dose levels. Hereby, 100 kVp images and different levels of VMI were evaluated regarding objective and subjective image criteria. Furthermore, the radiation exposure of both systems should be evaluated in a large number of patients.

## 2 Material and methods

### 2.1 Phantom study

A semi-anthropomorphic thorax phantom (QRM-Abdomen, QRM GmbH, Moehrendorf, Germany) was used. The phantom contains a central borehole with an insert for up to eight rods. In the present study, rods contained iodine concentrations of 0.5, 0.75, 1, 2, 5, 10 and 15 mg/mL as well as one rod with water density. The phantom has axial diameters of 300 × 200 mm, a length of 200 mm and was equipped with one extension ring, resulting in a total axial diameter of 350 × 250 mm.

The phantom was scanned (IQon Spectral CT, Philips Healthcare, The Netherlands) using a CTDI_vol_ of 4.85 mGy (which was the mean CTDI_vol_ for all patients at the timepoint of the reader study) with tube voltages of 80, 100 and 120 kVp, respectively. Tube current was adjusted to maintain the respective CTDI_vol_ at the given tube voltage with values of 162 mAs (80 kVp), 85 mAs (100 kVp) and 54 mAs (120 kVp). Every scan was repeated three times and in-between scans, the phantom was repositioned. For 120 kVp, monoE-40, monoE-50, monoE-60 and monoE-70 images were calculated. All images were reconstructed with a slice thickness of 1.0 mm.

Regions of interest (ROI) with an area of 1 cm² were analyzed in 3 different slices for each iodine concentration in each scan and for each image type. In total, 486 ROIs resulted. For each ROI, intensity value was measured in Hounsfield Units (HU). For each ROI, CNR was calculated by: $\frac{S_{I}-S_{W}}{\delta_{W}}$, where S_I_ is the intensity measured in the iodine rod, S_W_ is the intensity of the water rod and δ_W_ is the standard deviation measured in the water rod.

### 2.2 Patient population

Institutional review board (IRB) approval was obtained for this retrospective study including all protocols (Technical University of Munich, School of Medicine, Ethics Commission). Informed consent was waived by the IRB as no additional data besides clinical obtained images were used. All examinations were performed exclusively for diagnostic use and were performed only with clinical standard protocols. All patient data were completely anonymized at the beginning of the study.

From November 1st, 2017 until December 31st, 2019, 2110 patients with suspected PE were examined. Imaging data of 60 patients (27 men, 33 women) was selected retrospectively for the imaging analysis. Hereby, the data was selected to match similar average radiation doses for C-CT and SD-CT. At the timepoint of the beginning of the reader study, the man CTDI_vol_ for all patients with suspected PE as 4.85 mGy. So, this dose level was selected as the sighted mean dose level of the reader study patients as well. Patients were selected without knowledge of the respective images to avoid any bias. Patients were examined using a conventional spiral CT-system (C-CT: Brilliance iCT, Philips Healthcare, The Netherlands) or a spectral-detector CT-system (SD-CT: IQon Spectral CT, Philips Healthcare, The Netherlands). For C-CT, only imaging data of examinations with a tube voltage of 100 kVp was used. For SD-CT, spectral information was obtained from spectral base image (SBI) datasets (spectral-dedicated DICOM files). Mean age for patients scanned with C-CT was 59.4 years (27–86) and 65.9 years (33–95) for patients scanned with SD-CT. PE was classified as central, segmental or subsegmental; PE in multiple locations was also possible (Table 1).

**Table 1:** Prevalence of pulmonary embolism (ranked as central, segmental or subsegmental) for the patients included into the reader study. For both CT-systems, a total of 15 patients with pulmonary embolism were included. PE in multiple locations was possible, resulting in the higher number of total locations with PE.

	central	segmental	subsegmental
C-CT	4	12	15
SD-CT	2	12	14

For the reference standard, the clinical report as well as a repeated reading of the images by the author was used. Examinations not suitable for clinical evaluation (i.e. due to contrast phase or motion artefacts) were excluded based on the clinical report. Also, examinations with other severe findings (e.g. large pleural effusion, pulmonary mass, atelectasis) were excluded to minimize recall bias and to focus on the image quality regarding diagnosis of PE.

### 2.3 CT parameters

The same scanning protocol was used for both CT-systems. Scans were performed with 60 mL of intravenous contrast agent (Imeron 400, Bracco Imaging Deutschland GmbH, Konstanz, Germany) followed by a 50 mL saline chaser with an injection rate of 3.5 mL/s. The scan range covered the whole thorax, was performed in inspiration and was chosen by an anteroposterior scout. The bolus tracker was placed within a region-of-interest (ROI) in the pulmonary trunk (threshold for scan start: 100 HU). The scan was performed craniocaudally with a pitch of 0.9 and a 128 × 0.625-mm (C-CT) or 64 × .0.625 mm (SD-CT) detector coverage. Obese patients were examined using a tube voltage of 120 kVp for both systems. Non-obese patients were examined with 120 kVp for SD-CT compared to 100 kVp for C-CT. CTDI_vol_ was selected via automatic dose control for all patients (C-CT and SD-CT). A tube voltage of 100 kVp corresponds to a mean energy of ∼60 keV and a tube voltage of 120 kVp corresponds to a mean energy of ∼70 keV.

Tomographic slices were obtained with a field of view of 350−500 mm, based on the diameter of the patient. A 512 × 512 image matrix with a slice thickness of 0.9 mm was used for axial thin slices data.

### 2.4 Dose information and image calculation

For every patient, the automatically generated dose protocol was extracted after the examination. Tube voltage (kVp), tube current (mA), volume-weighted CT dose index (CTDI_vol_) and dose length product (DLP) were collected. By multiplication of DLP by the chest conversion factor (0.0145), the effective dose (ED) could be calculated [37].

For SD-CT examinations, virtual monoenergetic images (VMI) with 40 keV, 60 keV and 70 keV were calculated using the commercially available spectral workstation (IntelliSpace Portal (v. 9.0), Philips Healthcare, The Netherlands). Thus, the following datasets were obtained:

1) 100 kVp using C-CT.

2) monoE-40 (VMI with 40 keV), monoE-50 (VMI with 50 keV), monoE-60 (VMI with 60 keV) and monoE-70 (VMI with 70 keV, approximately corresponding to 120 kVp and thus the standard clinical data) using SD-CT.

### 2.5 Subjective image quality

Each dataset was independently evaluated by three blinded radiologists (board certified, 4.0 ± 1.0 years of experience), the author not being a reader. To avoid a recall bias, only 30 cases were evaluated at a single timepoint with a minimum of two weeks between the sessions. Cases were presented in a randomized order, i.e. case number and type of dataset - C-CT/monoE-40/monoE-60/monoE-70 - was randomized. Image parameters (slice thickness, window level and width) could be individually chosen by the readers. Subjective image quality was rated regarding the following criteria:

1- not diagnostic; 2 - sufficient; 3 - satisfactory; 4 - good; 5 - very good; 6 - excellent

Contrast of pulmonary vessels was rated. Hereby, central pulmonary vessels (pulmonary trunk and main pulmonary arteries), segmental pulmonary arteries and subsegmental pulmonary arteries were analyzed. For subsegmental arteries, four different regions were rated (upper and lower zone for the right and left lung, respectively) to avoid influence due to a partially decreases/increased contrast in singular subsegmental vessels. Contrast was rated using the following scale:

1 - not diagnostic; 2 - sufficient; 3 - satisfactory; 4 - good; 5 - very good; 6 - excellent

The presence of streaking artefacts was assessed using the classes:

1 - massive artefacts (image not diagnostic); 2 - major artefacts; 3 - minor artefacts; 4 - no artefacts.

### 2.6 Diagnostic confidence regarding detection of PE

Diagnostic confidence regarding detection of PE was evaluated for each localization (central, segmental, subsegmental) separately. Hereby, confidence was rated using the following scale:

1 - no PE, completely confident; 2 - no PE, probably confident; 3 - no PE, poor confidence, additional imaging needed; 4 - PE present, poor confidence, additional imaging needed; 5 - PE present, probably confident; 6 - PE present, completely confident. Ratings of 1–3 were considered negative for PE (PE_N_) and ratings of 4–6 as positive for PE (PE_P_). PE should only be rated as positive in the specific location when a clear embolus was visible.

### 2.7 Statistical analysis

Statistical analysis was performed by dedicated software packages (SPSS, IBM, USA; Excel 2016, Microsoft, USA; Prism 7, Version 7.0c). Continuous data are expressed as arithmetic mean ± SD. Data were tested for Gaussian distribution via D'Agostino-Pearson omnibus test. As Gaussian distribution was present, two-sided paired *t*-test was used for comparison of CNR values and dose information, respectively. Statistical evaluation of subjective image criteria between different VMI-levels was performed using Wilcoxon signed-rank test as paired samples were present. Differences between different CT-systems were compared using Mann-Whitney-test as samples were not paired. For all test, a p-value ≤ 0.05 was considered to indicate statistical significance. Interobserver agreement was evaluated using Fleiss’ kappa. Diagnostic confidence was determined using sensitivity, specificity and accuracy.

## 3 Results

### 3.1 Phantom study - CNR

CNR in the phantom study was significantly higher in monoE-40 compared to other VMI and to C-CT (Table 2). CNR was lower for higher tube voltages and higher monoE-levels, respectively. There was no significant difference between 100 kVp and monoE-60.

**Table 2:** CNR for different tube voltages and iodine concentrations. CTDI_vol_ was 4.85 mGy for all scans. Tube current (mAs) was adapted to keep CTDI_vol_ constant at the different tube currents.

tube voltage	tube current	iodine concentration (mg/mL)						
		0.5	0.75	1.0	2.0	5.0	10	15
C-CT: 80 kVp	162 mAs 4.85 mGy	29.27 ± 1.29	37.53 ± 1.63	48.15 ± 0.91	92.54 ± 2.11	290.88 ± 1.03	360.46 ± 1.64	639.15 ± 3.66
C-CT: 100 kVp	85 mAs 4.85 mGy	26.10 ± 0.24	33.02 ± 0.28	44.63 ± 1.80	75.21 ± 1.16	229.61 ± 1.17	284.58 ± 0.51	501.39 ± 1.31
SD-CT: 120 kVp	54 mAs 4.85 mGy	23.01 ± 1.47	28.47 ± 0.74	35.24 ± 1.19	63.29 ± 0.66	187.93 ± 1.81	233.84 ± 1.21	409.62 ± 0.83
monoE-40	54 mAs 4.85 mGy	42.89 ± 2.30	68.92 ± 2.16	93.02 ± 2.10	178.35 ± 3.5	578.85 ± 2.98	724.2 ± 5.19	1290.8 ± 7.63
monoE-50	54 mAs 4.85 mGy	31.93 ± 0.61	46.15 ± 1.82	63.91 ± 1.99	114.4 ± 3.32	277.74 ± 1.40	556.78 ± 2.46	822.86 ± 4.10
monoE-60	54 mAs 4.85 mGy	24.05 ± 0.85	33.96 ± 0.61	44.30 ± 0.37	80.94 ± 0.54	252.31 ± 1.01	313.65 ± 1.89	554.19 ± 2.3
monoE-70	54 mAs 4.85 mGy	19.87 ± 0.95	26.3 ± 0.26	33.63 ± 0.17	59.5 ± 0.16	179.46 ± 1.69	222.33 ± 1.29	390.15 ± 1.12

### 3.2 Dose information

From 11/01/2017 until 12/31/2019, 271 patients were examined using the C-CT and 1839 patients were examined using the SD-CT. Of those patients, 88 (C-CT) and 534 (SD-CT) were obese. Mean CTDI_vol_ was higher for C-CT (5.91 mGy) compared to SD-CT (5.23 mGy). For all scans with 100 kVp (non-obese patients), mean CTDI_vol_ was 4.60 mGy for C-CT whereas it was 4.35 mGy for all non-obese patients with SD-CT (Table 3). Each comparison between C-CT and SD-CT (all/non-obese/obese patients) showed a significantly lower radiation exposure with SD-CT.

**Table 3:** Dose information of both CT-systems (C-CT with 100 kVp and SD-CT with 120 kVp). Given are dose information for all patients of one system combined (obese and not obese) as well as separated information for both groups. Significant differences (p < 0.05) were found for each comparison between C-CT and SD-CT (all, non-obese, obese).

CT system	obese	kVp	mAs	CTDI_vol_ (mGy)	DLP (mGy*cm)	ED (mSv)
C-CT	all	100/120	263.2 ± 90.6	5.69 ± 2.83	233.0 ± 112.0	3.38 ± 1.62
SD-CT	all	120	169.7 ± 98.7	5.14 ± 3.03	191.4 ± 115.1	2.77 ± 1.67
C-CT	no	100	262.6 ± 72.9	4.65 ± 1.28	193.3 ± 56.3	2.80 ± 0.82
SD-CT	no	120	141.1 ± 69.7	4.30 ± 2.10	161.4 ± 81.2	2.34 ± 1.18
C-CT	yes	120	264.3 ± 120.0	7.87 ± 3.77	315.4 ± 148.9	4.57 ± 2.16
SD-CT	yes	120	222.8 ± 122.2	6.80 ± 3.80	250.1 ± 145.4	3.63 ± 2.11

The CTDI_vol_ of the selected patients in the reader study was 4.85 mGy for both C-CT and SD-CT. For C-CT, a standard deviation of 1.22 mGy (range 3.3–8.1 mGy) was found, whereas for SD-CT the standard deviation was 1.74 mGy (range 3.2–9.7 mGy).

### 3.3 Subjective image quality

**Fig. 1:**
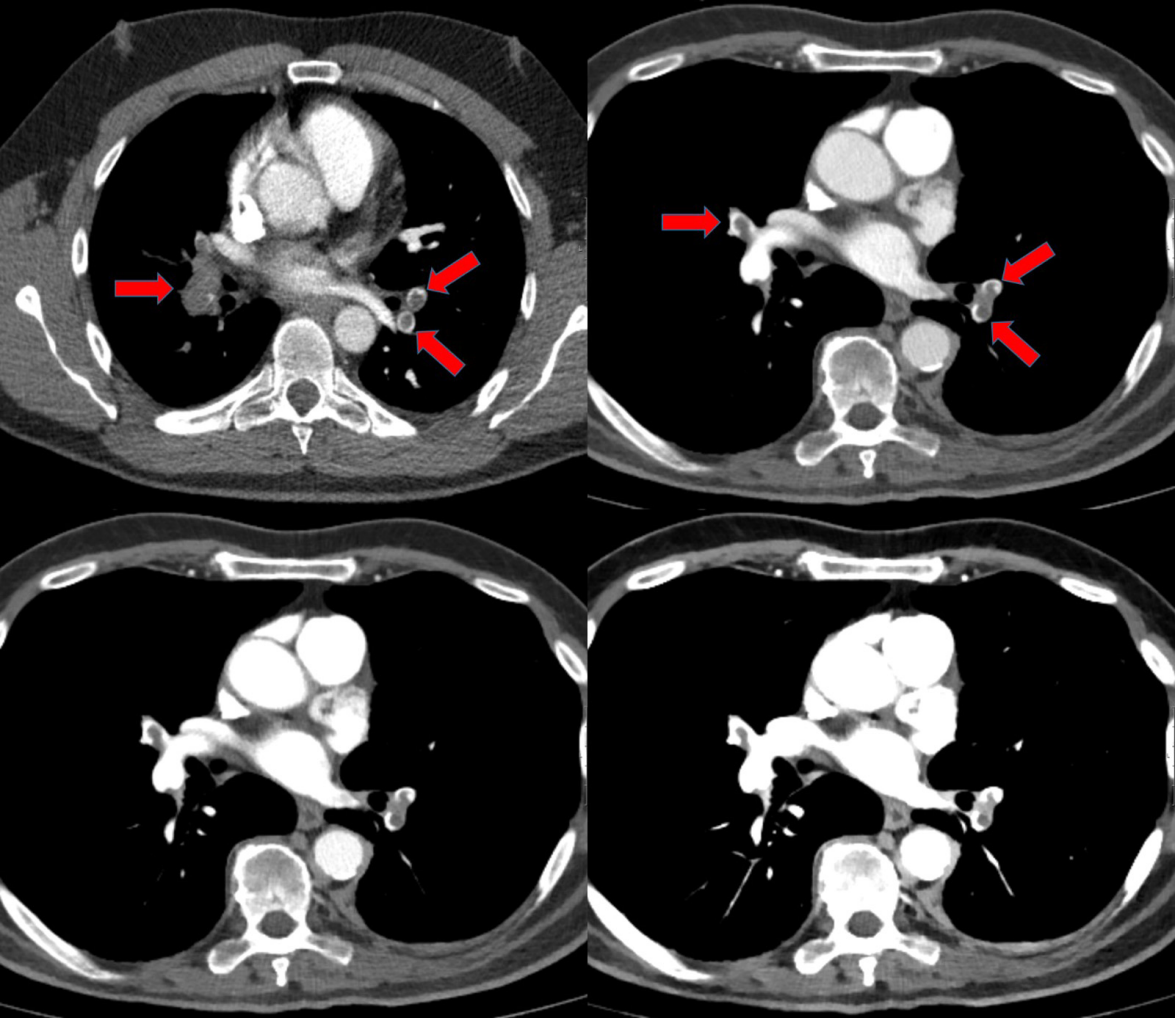
Comparison of two comparable patients scanned with the C-CT at 100 kVp (top left) and with the SD-CT at 120 kVp (top right and bottom). VMI of 70 keV (top right), 60 keV (bottom left) and 40 keV (bottom right) are shown. Pulmonary emboli are marked with red arrows. Note the clearly increasing contrast towards lower VMI-levels. As all images are shown at the same window setting (center: 100 HU, width 600 HU), VMI of 40 keV show a saturated iodine signal (For interpretation of the references to colour in this figure legend, the reader is referred to the web version of this article).

A comparison of images obtained with the C-CT (100 kVp) and with the SD-CT (monoE-40, monoE-60 and monoE-70) is shown in Fig. 1.

Subjective contrast level of pulmonary vessels was highest in monoE-40 images for all subsections; however, lowest subjective image quality was shown for those image type. Highest subjective image quality was shown for monoE-60 and monoE-70 images with significantly higher image quality compared monoE-40 and 100 kVp (Table 4). No significant difference in the rating of artefacts were found at any subgroup. Hereby, the best value was found for monoE-70 (mean: 3.17) and the worst value (mean: 3.02) was found for monoE-40. Interobserver agreement was low for each comparison (total, C-CT, VMIs) and was not clearly higher for one dataset, apart from central contrast for monoE-40 (Table 5). Here, a full agreement (k = 1) was found as each case was rated with ‘excellent contrast’.

**Table 4:** Subjective image quality of the examined images. Additionally, subjective contrast in central, segmental and subsegmental pulmonary arteries is given. All ratings regarding image quality and contrast were performed based on a 6-point scale. Artefacts were rated on a 4-point scale.

	monoE-40	monoE-60	monoE-70	100 kVp
image quality	4.72 ± 0.46^^* x^^	5.48 ± 0.42^+^	5.43 ± 0.51 ^+^	4.87 ± 0.53
contrast central	6.00 ± 0.00^^+ x^^	5.92 ± 0.19^^x +^^	5.54 ± 0.48 ^+^	5.12 ± 0.4
contrast segmental	5.73 ± 0.22^*x +^	5.59 ± 0.36^^x +^^	5.22 ± 0.60 ^+^	4.49 ± 0.91
contrast subsegmental	5.51 ± 0.25^^* x +^^	5.13 ± 0.43^^x +^^	4.55 ± 0.55 ^+^	3.68 ± 0.66
artefacts	3.02 ± 0.30	3.11 ± 0.36	3.17 ± 0.48	3.06 ± 0.47

*Significance to monoE-60.

^x^significance to monoE-70.

^+^significance to 100 kVp.

**Table 5:** Interobserver agreement, given in Fleiss' kappa.

	total	C-CT	monoE-40	monoE-60	monoE-70
Image quality	0.09	−0.06	−0.15	0.07	0.16
contrast central	0.17	−0.03	1.00	0.22	0.00
contrast segmental	−0.04	−0.06	−0.24	−0.16	−0.06
contrast subsegmental	0.09	−0.15	−0.11	0.00	−0.02

### 3.4 Diagnostic performance

Diagnostic confidence was high or medium in most cases when using monoE-40 images (Table 6). As expected, diagnostic confidence was higher for central PE than for segmental or subsegmental PE.

**Table 6:** Shown are the mean number of cases with high/medium/low confidence regarding the diagnosis of central/segmental/subsegmental pulmonary embolisms.

	central			segmental			subsegmental		
	high	med.	low	high	med.	low	high	med.	low
monoE-40	29.0	0.33	0.67	26.0	2.67	1.33	21.0	6.67	2.33
monoE-60	28.7	0.33	1.00	27.0	2.33	0.67	19.7	7.33	3.00
monoE-70	28.0	1.67	0.33	24.0	3.67	2.33	18.7	6.33	5.00
100 kVp	26.0	2.33	1.67	23.3	1.33	5.33	13.7	6.00	10.3

**Fig. 2:**
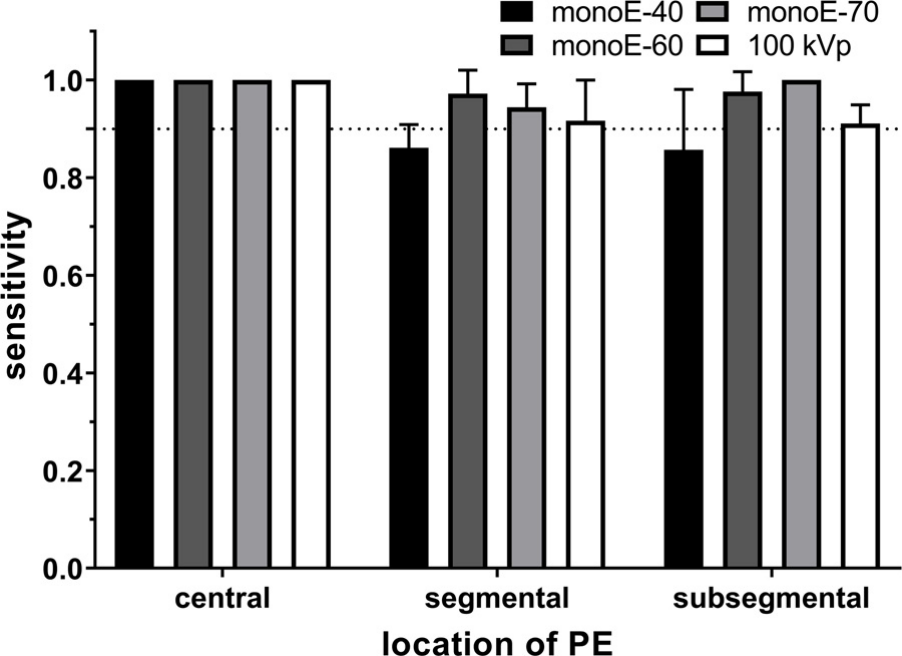
Sensitivity (mean + SD of all readers) for the different image types, broken down for the different locations of PE.

Sensitivity was higher than 90 % for all locations of PE for all image types, except monoE-40 (Fig. 2). For central PE, sensitivity was 100 % for all image types. With monoE-60, sensitivity was greater than 97 % for segmental and subsegmental PE, respectively.

Specificity was higher than 90 % for all image types and locations of PE, except for subsegmental PE at 100 kVp (Table 7). For accuracy, highest values were again found for monoE-60 and monoE-70 images. For these image types, accuracies higher than 95 % were found for all locations of PE.

**Table 7:** Specificity and accuracy for central, segmental and subsegmental PE.

	specificity			accuracy		
	central	segmental	subseg.	central	segmental	subseg.
monoE-40	0.95 ± 0.02	0.94 ± 0.06	1.00 ± 0.00	0.96 ± 0.02	0.91 ± 0.02	0.93 ± 0.06
monoE-60	0.99 ± 0.02	0.98 ± 0.03	0.96 ± 0.07	0.99 ± 0.02	0.98 ± 0.02	0.97 ± 0.06
monoE-70	0.95 ± 0.05	0.98 ± 0.03	0.96 ± 0.04	0.96 ± 0.05	0.97 ± 0.00	0.98 ± 0.02
100 kVp	0.94 ± 0.02	0.93 ± 0.03	0.80 ± 0.12	0.94 ± 0.02	0.92 ± 0.02	0.86 ± 0.04

## 4 Discussion

In this work, conventional CT scans obtained at 100 kVp were compared to virtual monochromatic images calculated from spectral data obtained with a spectral detector CT at 120 kVp. Both approaches were evaluated regarding objective and subjective image criteria using phantom scans and retrospectively collected in-vivo patient data. The results demonstrated that both methods enabled excellent diagnostics for detection of PE at an equivalent dose level. Hereby, SD-CT offers additional capabilities, including iodine quantification, reduced beam-hardening artifacts and increased iodine signal via low-keV VMI. Additionally, lower radiation doses were found for SD-CT compared to C-CT when evaluating over 2000 examinations.

In the current study, a sensitivity of 100 % for central PE could be reached with all VMI datasets as well as with C-CT. The best results regarding sensitivity and specificity of segmental and subsegmental PE could be found when monoE-60 and monoE-70 were used. For sensitivity, C-CT was superior to monoE-40, whereas the opposite was found for specificity. These results correspond with the results of subjective image quality where the best results were also reached with monoE-60 and monoE-70. This is most likely as in clinical routine, radiologists are used to these image types. As different patients were used for SD-CT and C-CT, the diagnostic performance of both systems cannot be compared to the full extend. A recall bias is thinkable as for monoE-40, monoE-60 and monoE-70, the same patient data were used. To minimize possible recall bias, readers were advised to evaluate a maximum of 30 cases during one reading session with a minimum of two weeks between two sessions. The order of cases was randomized, so that no VMI should be favored, even if a recognition of cases was present. Overall, the present results indicate that SD-CT is equivalent or even superior to C-CT regarding subjective image quality and diagnostic performance at an equivalent dose level. For image quality and subjective contrast, small standard deviations were found, indicating consistency in the respective groups and between the readers. Despite the small standard deviations, a low interobserver agreement was found. This is most likely due to a narrow range but not identical ratings and the use of Fleiss’ kappa, calculating the agreement of all three raters at once. However, given the small standard deviations, the results seem consistent and reliable.

The results of the diagnostic performance do not correlate to the diagnostic confidence and subjective contrast reported by the readers. This could be as radiologists correlate a high contrast to a high diagnostic confidence. However, a strong increase of the iodine signal does not seem to increase the diagnostic performance - at least in the current study where a sufficient contrast of the pulmonary vessels was present in all cases. Additionally, the diagnostic performance could be lower for monoE-40 due to a lack of experience with the datasets. Another factor for the diagnostic superiority of monoE-60 and monoE-70 compared to monoE-40 could be the examined patient type. As a 100 kVp protocol was used for C-CT, only non-obese patients could be included. As the radiation dose should be matched with SD-CT, obese patients were not included for this system as well. In obese patients, where image quality is degraded at low-kVp levels, monoE-40 could be beneficial due to the boost in the iodine signal. However, as there is an increased rate of photon starvation for low-energy photons, monoE-40 images could also show decreased image quality in obese patients. A main advantage of monoE-40 images is the possibility of a boost in the iodine signal when there is reduced contrast of the pulmonary vessels.

With conventional image reconstruction (i.e. filtered back projection) the image noise significantly increases with low tube voltages or low keV VMI settings. Our CNR measurements indicated that this noise related effect did not disrupt the boost in the iodine signal. Iterative reconstruction algorithms enable the dose efficient applicability of both modalities by suppressing the increased noise level [14,38]. This observation from phantom experiments translates to our reader study. Subjective image assessment reveals that image contrast is significantly improved at 40 keV VMI compared to higher VMI and low-kVp C-CT. At the same time, overall image quality was assessed highest for VMIs at 60 and 70 keV. The reason for this specific result may be the fact that those VMI settings offer improved contrast while image appearance is close to conventional CT.

In the current study, radiation exposure was significantly lower with SD-CT compared to C-CT for each comparison (all patients, non-obese patients, obese patients). This shows that despite concerns regarding radiation exposure, it may even be lower for SD-CT compared to conventional CT-systems. This partly might be due to improved protocols which were adapted for the new system. However, we constantly optimize our protocols of each system to minimize radiation exposure so that the observed reduced radiation exposure seems realistic. Certainly, SD-CT systems do not go along with an increased radiation exposure compared to C-CT, not even if a 100 kVp protocol is used for the latter.

DE-CT enables the generation of iodine maps for the assessment of the lung perfusion increasing the sensitivity for the detection of PE [39,40]. SD-CT - in contrast to other DE-CT systems - additionally enables the retrospective generation of spectral data, e.g. in cases of an impaired contrast to calculate low-keV VMI [41]. Consequently, repeated examinations can be avoided resulting in a decreased radiation exposure. Increased sensitivity, particular for subsegmental PE, may accelerate further diagnostic and therapeutic steps for the individual patient.

The presented study design is subject to limitations. Firstly, the performance and radiation dose levels are strongly depending on clinical applications. Our results are specific for PE diagnostics, and further studies are essential to evaluate other applications. Additionally, obese patients could not be evaluated as those are not examined using 100 keV. Further, different patients were used for the two CT systems. Despite matching dose levels and excluding non-diagnostic examinations, an influence of the patient selection to the results could be possible. In the current study, a combination of VMI and iodine maps was not evaluated. This combination could yield even higher sensitivity, specificity and diagnostic accuracy compared with VMI alone or compared to 100 kVp examinations. This evaluation should be subject of further studies. Despite the stated drawbacks the current study gives an essential indicator that either approach can be potentially employed with high diagnostic performance and without major drawbacks.

## 5 Conclusion

In conclusion, this study illustrates that detection of PE is enabled for non-obese patients at an equivalent diagnostic level and a similar radiation exposure with SD-CT compared to C-CT at 100 kVp. With SD-CT even reduced radiation doses were found for obese and non-obese patients. In the clinical setting where spectral results - such as material specific maps or VMI - are useful, SD-CT may be the modality of choice.

## Ethics approval and consent to participate

Institutional review board (IRB) approval was obtained for this retrospective study including all protocols (Technical University of Munich, School of Medicine, Ethics Commission). Informed consent was waived by the IRB as no additional data besides clinical obtained images were used. All examinations were performed exclusively for diagnostic use and were performed only with clinical standard protocols. All patient data were completely anonymized at the beginning of the study.

## Availability of data and materials

The datasets used and/or analyzed during the current study are available from the corresponding author on reasonable request.

## Funding

This work was supported by the German Department of Education and Research (BMBF) under grant IMEDO (13GW0072C), the German Research Foundation (DFG) within the Research Training Group GRK 2274 and the German Research Foundation (DFG) within the project 432290010. Funding had no influence on the results of this study.

## CRediT authorship contribution statement

**Andreas P. Sauter:** Conceptualization, Data curation, Formal analysis, Methodology, Supervision, Validation, Visualization, Writing - original draft. **Nadav Shapira:** Conceptualization, Methodology, Writing - original draft. **Felix K. Kopp:** Investigation, Software, Methodology, Writing - review & editing. **Juliane Aichele:** Investigation, Writing - review & editing. **Jannis Bodden:** Investigation, Writing - review & editing. **Andreas Knipfer:** Investigation, Writing - review & editing. **Ernst J. Rummeny:** Funding acquisition, Project administration, Writing - review & editing. **Peter B. Noël:** Funding acquisition, Methodology, Project administration, Validation, Writing - original draft.

## Declaration of Competing Interest

NS is an employee of Philips GmbH.

The remaining authors of this manuscript declare no relationships with any companies, whose products or services may be related to the subject matter of the article.
